# A Large Cardiac Metastasis of a Parathyroid Tumour Presenting with Ventricular Tachycardia

**DOI:** 10.5935/abc.20180255

**Published:** 2019-01

**Authors:** Rita Ilhão Moreira, Sílvia Aguiar Rosa, Ana Galrinho, Nuno Jalles Tavares, Rui Cruz Ferreira

**Affiliations:** 1 Hospital Santa Marta - Centro Hospitalar Lisboa Central - Cardiologia, Lisboa - Portugal; 2 Hospital CUF Infante Santo - Imagem, Lisboa - Portugal

**Keywords:** Carcinoma, Squamous Cell, Neoplasms Metastasis, Parathyroid Neoplasms, Arrhythmias, Cardiac, Diagnosis, Imaging, Echocardiography/methods, Neoplasms Metastasis/therapy

**Figure 1:**
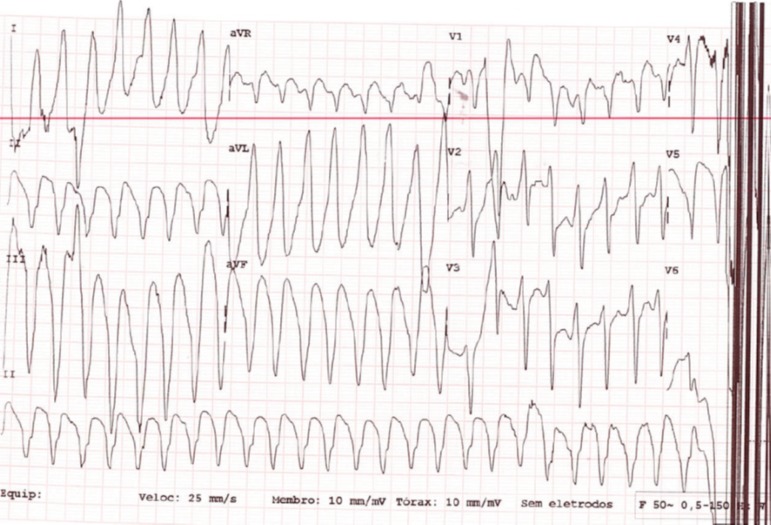
Twelve-lead electrocardiogram: Ventricular tachycardia with left bundle branch block morphology and superior and leftward axis consistent with a right ventricular origination of a tumour.

A-81-years old woman was admitted after an episode of ventricular tachycardia with hemodynamic instability converted after electrical cardioversion (Figure 1). Past medical history was significant for poorly differentiated squamous cell carcinoma of left parathyroid, diabetes and hypertension.

**Figure 2:**
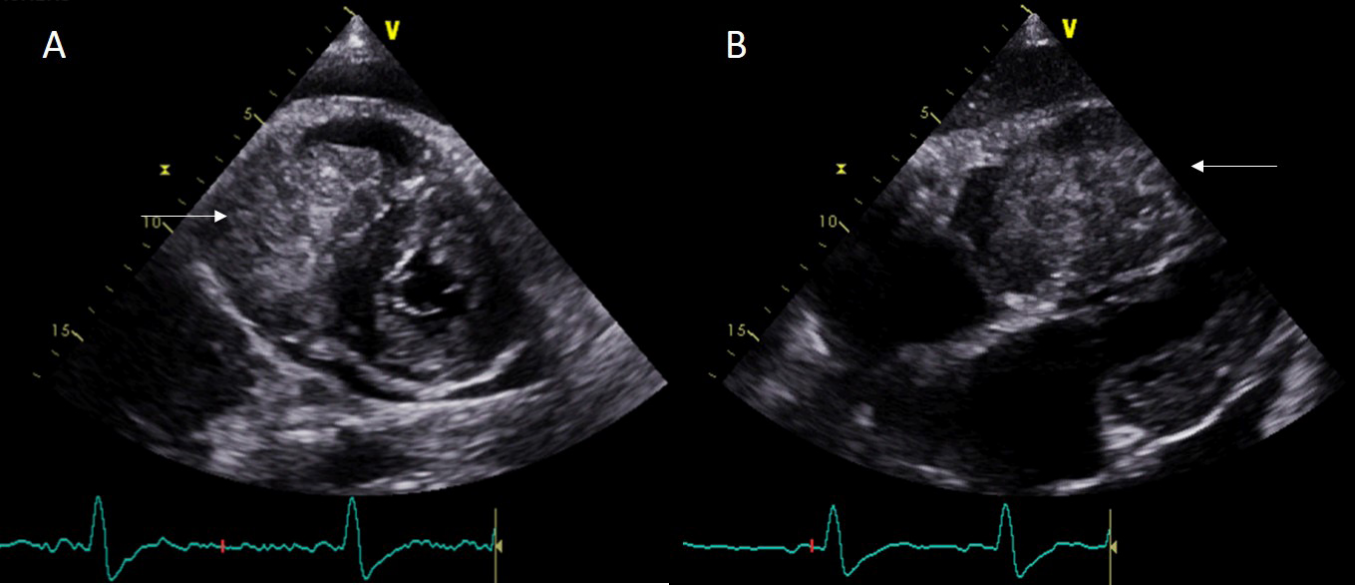
Transthoracic echocardiogram: Large mass in the right ventricle prolapsing into the right atrium in parasternal short axis view (panel A) and subcostal view (panel B). 230x99mm (150 x 150 DPI).

Echocardiogram revealed a large mass in the right ventricle prolapsing into the right atrium and a moderate pericardial effusion (Figure 2, Video 1).

**Video 1:**
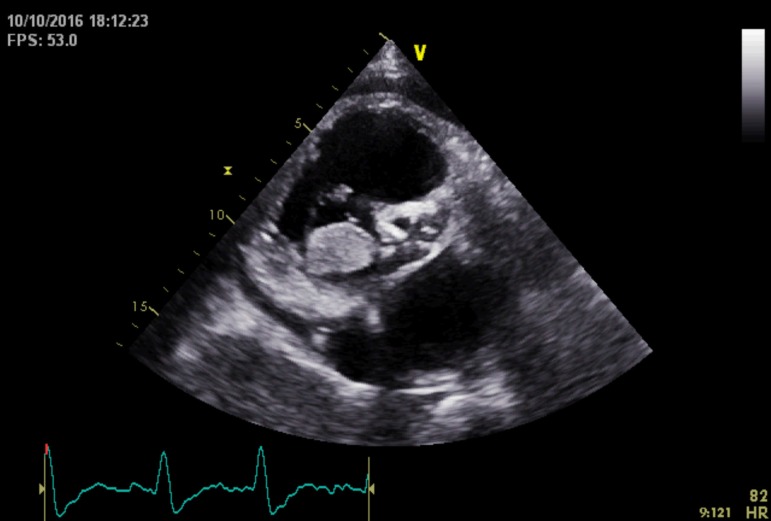
Echocardiogram revealed a large mass in the right ventricle prolapsing into the right atrium and a moderate pericardial effusion.

**Figure 3:**
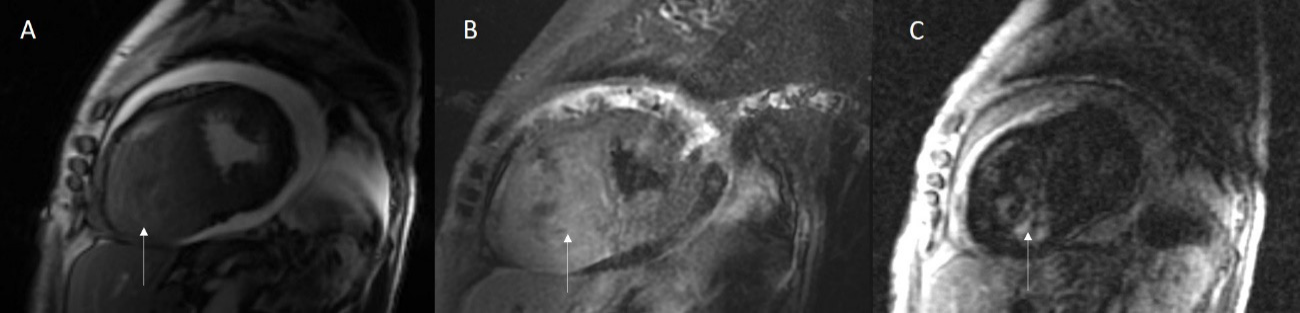
Cardiovascular Magnetic Resonance: Steady-state free precession imaging, in short axis view, documenting right ventricular mass (panel A); T2 weighted images showing mass with higher signal intensity compared to myocardium, in short axis view (panel B); Late gadolinium enhancement, acquired 10 minutes after gadolinium intravenous administration, showing a heterogeneous uptake of the mass, in short axis view (panel C). 328x78mm (150 x 150 DPI).

Cardiac magnetic resonance demonstrated a large infiltrative mass occupying almost the entire right ventricle cavity, slightly hypointense in T1 weighted images (image not available), hyperintense in T2 weighted images, with heterogeneous early and late gadolinium enhancement (Figure 3). These findings suggested cardiac sarcoma or metastasis.

On histopathological investigation performed with catheter biopsy, there were malignant cells positive for CK5/6 and p63 and negative for oestrogens consistent with a cardiac metastasis from a squamous cell carcinoma.

The primary malignancies most commonly metastasizing to the heart are breast cancer, lung cancer, leukaemia, and melanoma.^1^ Distant metastasis of head and neck tumours are highly unusual, especially of parathyroid.^2^ Generally, patients with distant metastases are considered to be inoperable, and only palliative treatments, such as chemotherapy or irradiation of a tumour, are indicated.^3^ Although infrequently, ventricular arrhythmia can be the initial presentation of a cardiac metastasis.^4^^,^^5^ We report a rare case of cardiac metastasis from a poorly differentiated squamous cell carcinoma of parathyroid presenting with ventricular arrhythmia.
